# Mitigation of Variability among 3D Echocardiography-Derived Regional Strain Values Acquired by Multiple Ultrasound Systems by Vendor Independent Analysis

**DOI:** 10.1371/journal.pone.0153634

**Published:** 2016-05-05

**Authors:** Cole Streiff, Meihua Zhu, Eriko Shimada, David J. Sahn, Muhammad Ashraf

**Affiliations:** Oregon Health & Science University, Portland, Oregon, United States of America; Scuola Superiore Sant'Anna, ITALY

## Abstract

**Introduction:**

This study compared the variability of 3D echo derived circumferential and longitudinal strain values computed from vendor-specific and vendor-independent analyses of images acquired using ultrasound systems from different vendors.

**Methods:**

Ten freshly harvested porcine hearts were studied. Each heart was mounted on a custom designed phantom and driven to simulate normal cardiac motion. Cardiac rotation was digitally controlled and held constant at 5°, while pumped stroke volume (SV) ranged from 30-70ml. Full-volume image data was acquired using three different ultrasound systems from different vendors. The image data was analyzed for longitudinal and circumferential strains (LS, CS) using both vendor-specific and vendor-independent analysis packages.

**Results:**

Good linear relationships were observed for each vendor-specific analysis package for both CS and LS at the mid-anterior segment, with correlation coefficients ranging from 0.82–0.91 (CS) and 0.86–0.89 (LS). Comparable linear regressions were observed for results determined by a vendor independent program (CS: R = 0.82–0.89; LS: R = 0.86–0.89). Variability between analysis packages was examined via a series of ANOVA tests. A statistical difference was found between vendor-specific analysis packages (p<0.001), while no such difference was observed between ultrasound systems when using the vendor-independent program (p>0.05).

**Conclusions:**

Circumferential and longitudinal regional strain values differ when quantified by vendor-specific analysis packages; however, this variability is mitigated by use of a vendor-independent quantification method. These results suggest that echocardiograms acquired using different ultrasound systems could be meaningfully compared using vendor-independent software.

## Introduction

Strain imaging is an increasingly prevalent method of quantifying myocardial mechanics. This technology is used to determine global and regional myocardial deformation throughout a cardiac cycle and is affiliated with cardiac magnetic resonance imaging (cMRI), cardiac computed tomography (CT) and echocardiography (echo)[1, 2]. Strain-based imaging techniques are predominantly used to monitor cardiac function and direct therapy under a variety of conditions such as myocardial infarction [3–6], mechanical dyssynchrony [7–10] and cardiomyopathy [11–18]. These strain-based methods provide a unique and sensitive approach to quantitatively express the function of the myocardium, which represents cardiac health [1–3, 5, 6, 9].

Speckle-tracking echocardiography (STE) derived strain has been validated both experimentally and clinically [19, 20]. Two-dimensional (2D) STE tracks the motion of natural acoustic markers between acquisition frames to detect the direction and magnitude of segmental wall motion. 2D STE is angle independent; however, it has been shown to be limited by through-plane motion and the complex architecture of the heart [21, 22].

Three-dimensional echocardiography (3DE) enables the measurement of myocardial deformation within a volumetric acquisition and is not subject to many of the limitations of 2D STE. The temporal resolution of 3DE suffers relative to that of 2D STE and there are concerns regarding the accuracy of 3DE-derived strain values [23, 24]. However, experimental and clinical studies have validated the strain determining capabilities of multiple vendor-specific analysis packages based on 3DE acquisitions [25–27].

It has been shown that, while comparable in accuracy to 2DE-based methods, 3DE-derived strain values acquired from different vendors’ ultrasound systems and analysis packages lack reproducibility [28, 29]. The variability in myocardial strain measurements of different analysis programs has not been extensively studied. Vendor-independent analysis packages, such as TomTec Image Arena (TomTec Imaging Systems, Unterschleissheim, Germany), provide a means by which 3D volumes from multiple ultrasound systems can be analyzed [30]. The strain values determined by TomTec Image Arena have been shown to accurately and reliably quantify myocardial deformation [31, 32].

Koopman *et al* showed that vendor independent software was capable of determining global strain values from B-mode images acquired using multiple ultrasound systems [33] while Risum *et al* confirmed this finding using 2D STE [34]. However, strain quantification from 3D volumes across ultrasound systems has not been adequately explored using vendor-independent software. This study aimed to establish the variability of circumferential and longitudinal strain values acquired from different vendors and demonstrate a reduced variability due to analysis using a vendor-independent analysis package.

## Materials and Methods

A closed-circuit pulsatile harvested pig heart model was designed for this study. Pig hearts were purchased from Carlton Farms (Carlton, Oregon), which processes pork for commercial sale and is licensed to provide organs for in vitro research. OHSU does not require IACUC approval for in vitro projects.

**Fig 1:**
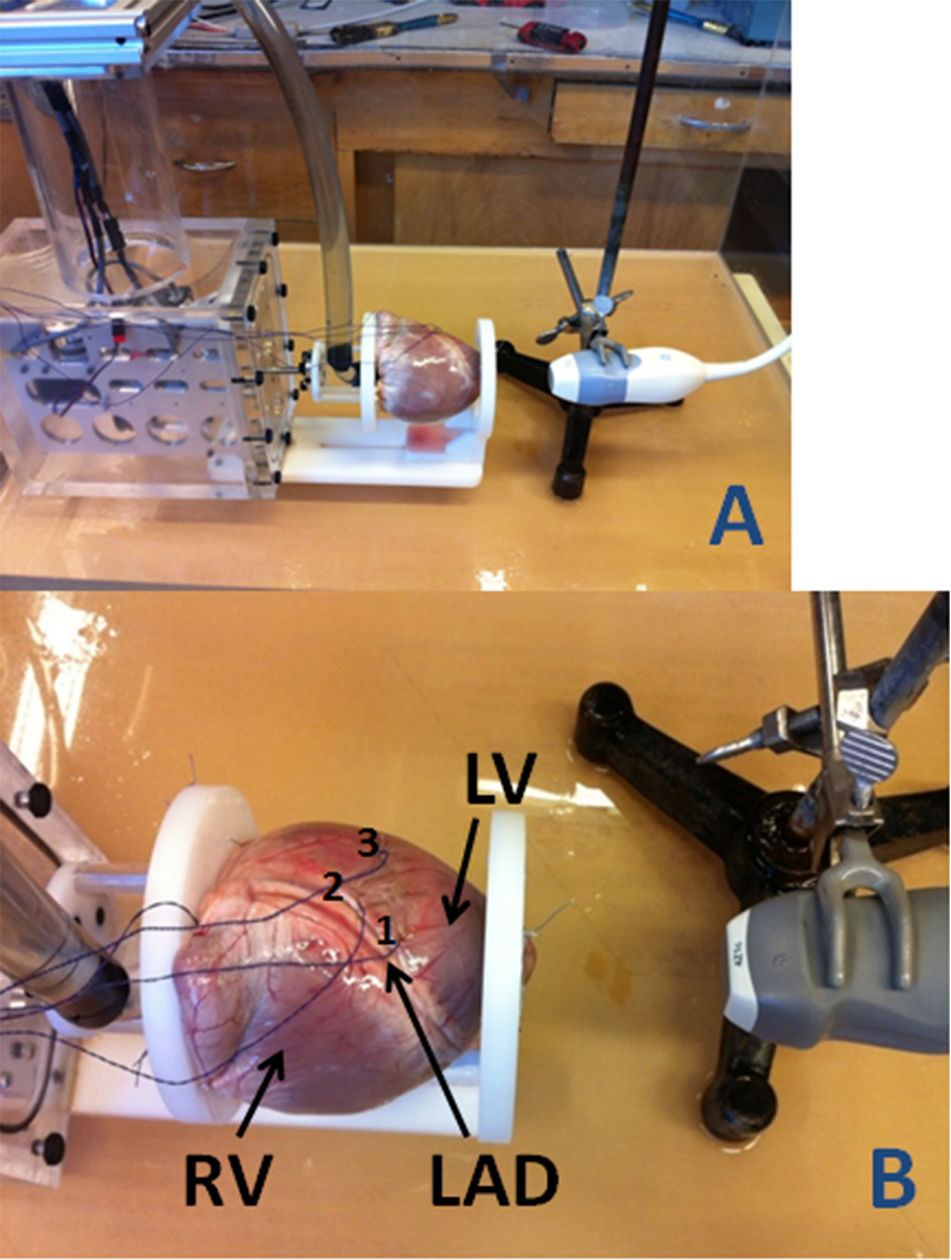
A) Experimental apparatus: Each heart is passively pumped in phase with a rotary actuator baseplate with images acquired from a fixed apex. B) Sonomicrometry crystal placement: Relative displacement between crystals 1 and 2 is used to determine reference longitudinal strain while displacement between crystals 2 and 3 represents reference circumferential strain.

Ten freshly harvested porcine hearts had atria and major vessels removed. The aortic leaflets were sutured together. A latex balloon was fixed within the left ventricle (LV) at the mitral annulus and connected to a pulsatile pump. Each phantom model was mounted on a custom mechanized base plate with the apex fixed (Fig 1A). The base plate was driven by a rotary actuator motor which was interfaced with the pulsatile pump and set to rotate 5° and compact 10mm in phase with the simulated cardiac cycle according to a simulated QRS Complex.

Three sonomicrometry crystals were sutured into the myocardial wall of the LV within the region adjacent to the left anterior descending coronary artery (LAD) (depicted in Fig 1B). The LAD was used as a landmark for the orientation of crystals numbered 1 and 2 which represented longitudinal strain (LS) and were perpendicular to crystal 3 which, coupled with crystal 2, provided circumferential strain (CS) reference values.

Each phantom was pumped at a stroke rate of 60 beats per minute (bpm) with a stroke volume (SV) ranging from 30-70ml. SV was increased in increments of 10ml with the appropriate volume added to the system to maintain a static end systolic volume (ESV). Full-volume 3DE data was acquired on the following ultrasound systems at each SV; GE Vivid E9 with a 3V-D transducer (GE Healthcare; Horten, Norway); Toshiba Aplio Artida with a PST-25SX transducer (Toshiba Medical System Corporation, Tochigi, Japan); and ACUSON SC2000 with a 4Z1c transducer (Siemens Medical Solutions USA Inc., Mountain View, CA). Each transducer was positioned in the same apical acquisition window and other parameters were conserved among ultrasound systems. The relative displacement between sonomicrometry crystals was recorded at each SV to provide reference strain values.

**Fig 2:**
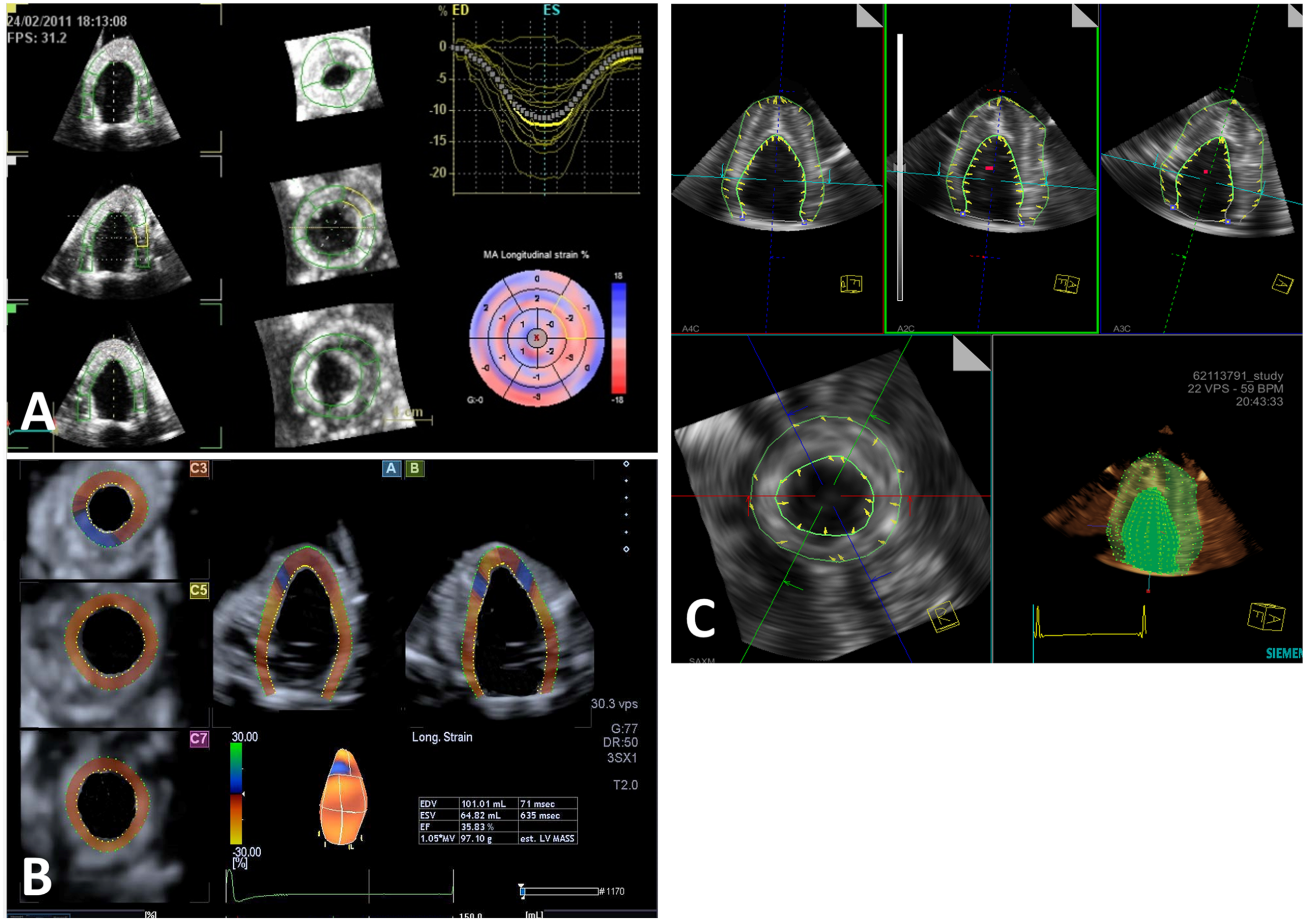
A) GE EchoPAC PC: Peak strain analysis using EchoPAC PC. B) Toshiba 3D-WMT: Peak strain analysis using Toshiba 3D-WMT. C) Siemens VMM: Peak strain analysis using Siemens VMM.

**Fig 3:**
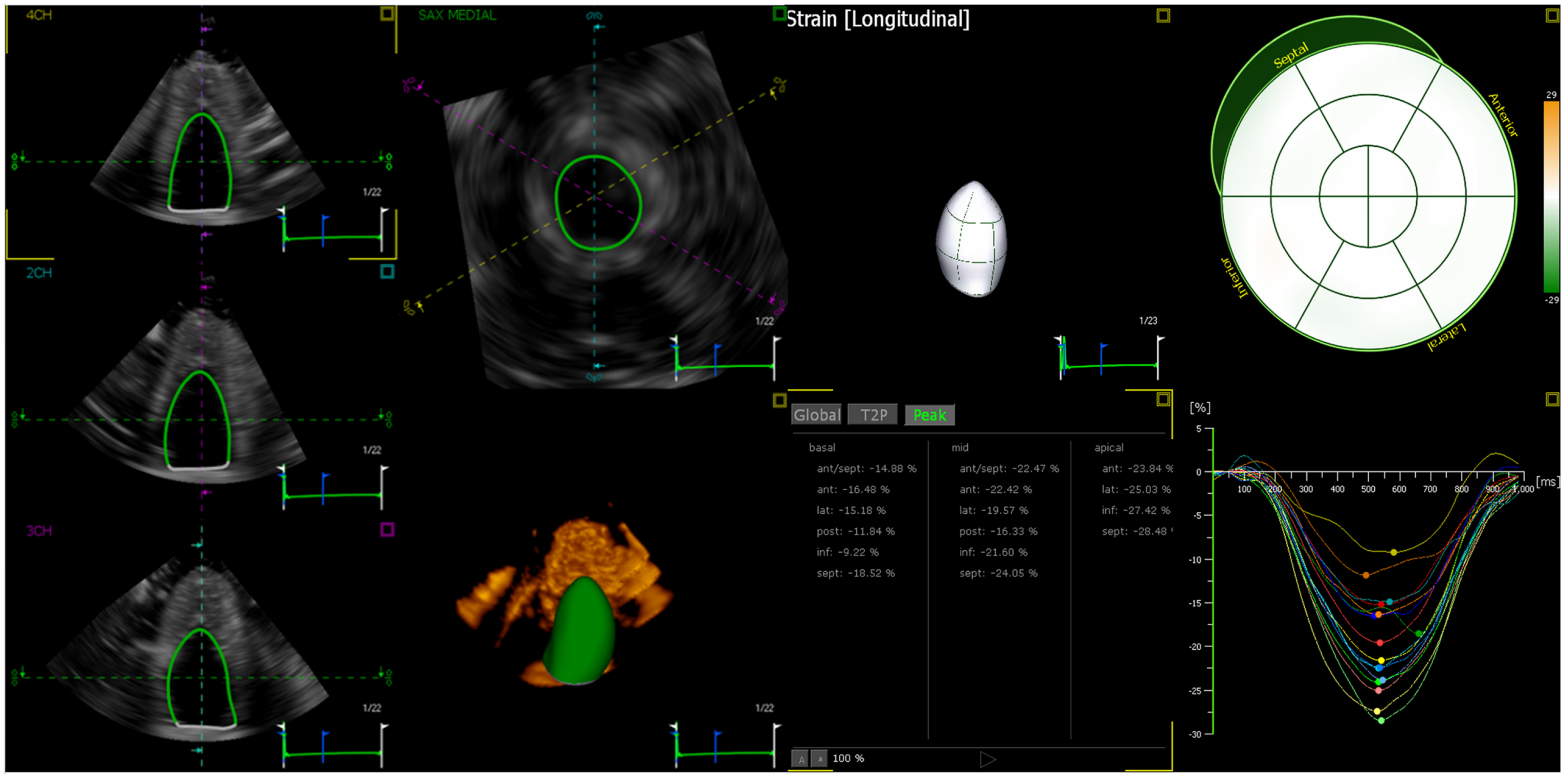
TomTec Image Arena: Peak strain analysis using TomTec Image Arena.

Data was analyzed offline by vendor-specific analysis packages: GE EchoPAC PC; Toshiba 3D-WMT; and Siemens VMM (Fig 2). Three isolated observers, each experienced with one of these analysis packages, obtained peak negative circumferential and longitudinal strain (CS and LS) values at the mid anterior (MA) segment of the LV for each SV of each phantom. The volumes used for each vendor-specific analysis were then stripped of identifying information and exported into TomTec Image Arena. Each observer analyzed a complete dataset using TomTec Image Arena to determine CS and LS at the MA for each volume (Fig 3). The sonomicrometry-derived displacement values were analyzed using SonoVIEW (SonoMetrics Corporation, Ontario, Canada) to generate reference CS and LS values at the MA segment.

Inter-observer variability was determined from a sample of 20% of the volumes analyzed by a different user blinded to the previously determined values. Intra-observer reproducibility was examined according to a sample of 20% of the volumes analyzed by the blinded initial operator six weeks after initial analysis. The intraclass correlation coefficients (ICCs) were calculated and presented.

## Results

Ten hearts were imaged by three distinct ultrasound systems at five different stroke volumes, resulting in a total of 150 full-volume acquisitions analyzed. Negative peak CS and LS were determined from each of these volumes using both vendor-specific and vendor-independent analysis programs. Including reference sonomicrometry results, over 750 data points were generated and used to determine the results presented in this study. The following data was excluded from all analysis due to inconsistent border tracking: Siemens Heart 4 CS SV 30-70ml and EchoPAC Heart 2 LS SV 40ml.

**Fig 4:**
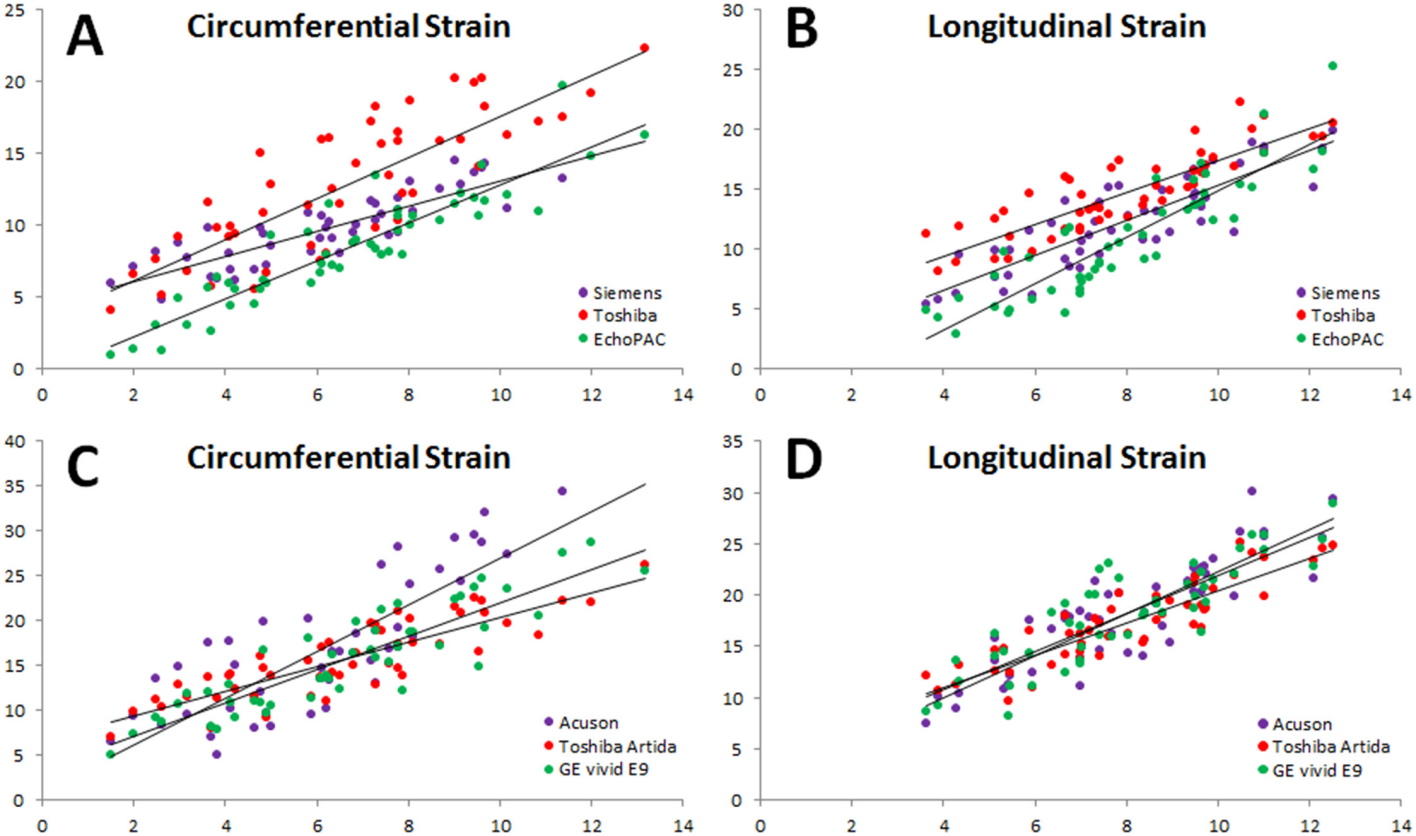
A) Circumferential strain derived from vendor specific analysis. B) Longitudinal strain derived from vendor specific analysis. C) Circumferential strain derived from vendor independent analysis. D) Longitudinal strain derived from vendor independent analysis. All echocardiography-derived strain values (y-axis) are plotted against sonomicrometry derived strain values (x-axis).

Circumferential and longitudinal strain data were analyzed separately. Linear regression analyses were performed on vendor specific echo-derived CS and LS values against sono-derived strain. The echo-determined strain values for GE EchoPAC PC, Toshiba 3D-WMT and Siemens VMM were plotted on the same xy coordinate plane against sonomicrometry determined strain (Fig 4A and 4B). Good linear agreement was observed for all analysis packages (CS: R = 0.82–0.91; LS: R = 0.86–0.89). Bias was gauged by a series of Bland-Altman analyses (CS: 1.8–6.1%; LS: 2.9–6.7%). Inter-vendor variability was determined significant for both CS and LS by one-way analysis of variance (ANOVA) tests (Table 1) with ICC values ranging from 0.52–0.66 (CS) and 0.59–0.77 (LS).

**Table 1:** One-way ANOVA results: Significant difference in vendor dependent CS and LS values among ultrasound systems and no significant difference in vendor independent results among ultrasound systems.

	ANOVA				
		P-value	F	F-*crit*	F > F-*crit*
Vendor-Dependent Analysis Package	Circumferential Strain	5.04E-12	31.41741	3.059831	Yes
	Longitudinal Strain	1.8E-15	43.22723	3.05805	Yes
Vendor-Independent Analysis Package	Circumferential Strain	0.060119	2.867829	3.059831	No
	Longitudinal Strain	0.361086	1.025779	3.05805	No

CS and LS values were determined by a vendor independent analysis package (TomTec Image Arena) and plotted in distinct datasets, based on the ultrasound system upon which the image loops were acquired, on the same xy coordinate plane against sonomicrometry determined strain (Fig 4C and 4D). Linear regression analyses were performed for each dataset and a good linear correlation was observed for all ultrasound systems (CS: R = 0.82–0.89; LS: R = 0.86–0.89). A series of Bland-Altman analyses revealed bias values for each dataset of 9.1–10.9% (CS) and 9.3–10.2% (LS). Inter-vendor variability was determined not to be significant by one-way analysis of variance (ANOVA) tests (Table 1) with ICC values ranging from 0.71–0.87 (CS) and 0.82–0.90 (LS). The variability of strain measurements computed from vendor specific and vendor independent analyses was determined by intra-class correlation (ICC). The ICC values using 1000 bootstrap samples are 0.5457392 (95% bootstrap CIs: [0.4445, 0.6390]) for vendor specific, and 0.793742(95% bootstrap CIs: [0.7182, 0.8465]) for vendor independent strain measurements. The lower bound of the 95% CI for ICC with vendor independent is higher than the upper bound of the 95% CI for ICC with Vendor specific. Higher intra-class correlation (ICC) indicates smaller inter-variability.

**Fig 5:**
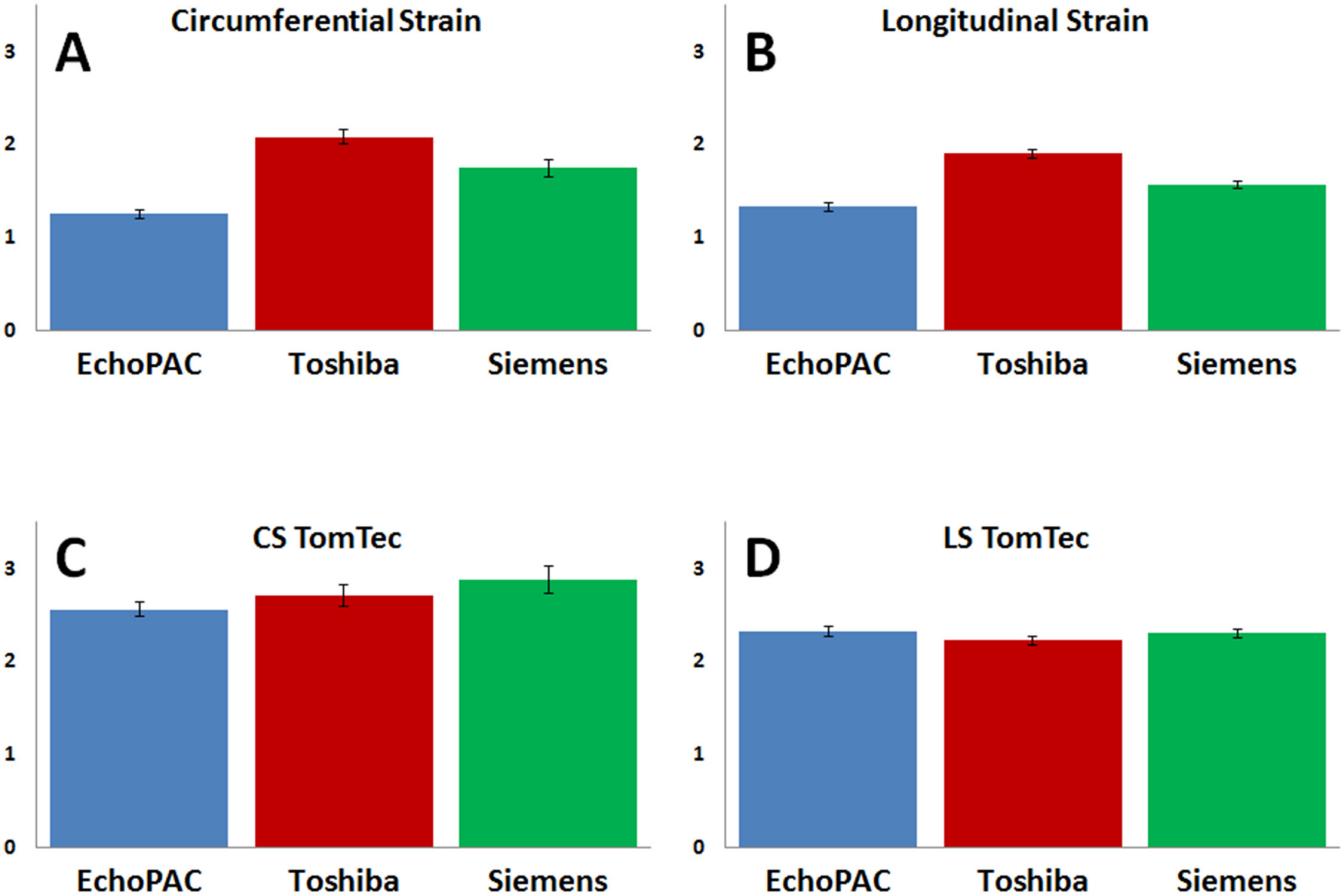
A) Normalized circumferential strain values from vendor specific analysis. B) Normalized longitudinal strain values from vendor specific analysis. C) Normalized circumferential strain values from vendor independent analysis. D) Normalized longitudinal strain values from vendor independent analysis. All error bars are represented as the standard error of the mean (SEM).

For standardization across SVs, CS and LS values were normalized by dividing the echo-derived strain by the reference strain. Normalized strain values were averaged across all hearts and SVs for each vendor-specific and -independent analysis package. These averages were plotted against each other with the error being represented by the Standard Error of the Mean (SEM). These normalized strain values were used to compare the vendor-specific derived CS and LS (Fig 5A and 5B) as well as the vendor-independent CS and LS (Fig 5C and 5D).

Analysis of Variance (ANOVA) tests were used to compare the difference of the strain values acquired from each vendor dependent and independent analysis package (Table 1). A statistical difference was found between vendor-specific analysis packages (F>F_crit_; p<0.001), while no such difference was observed between ultrasound systems when using the vendor-independent program (F<F_crit_; p>0.05) for both CS and LS.

**Fig 6:**
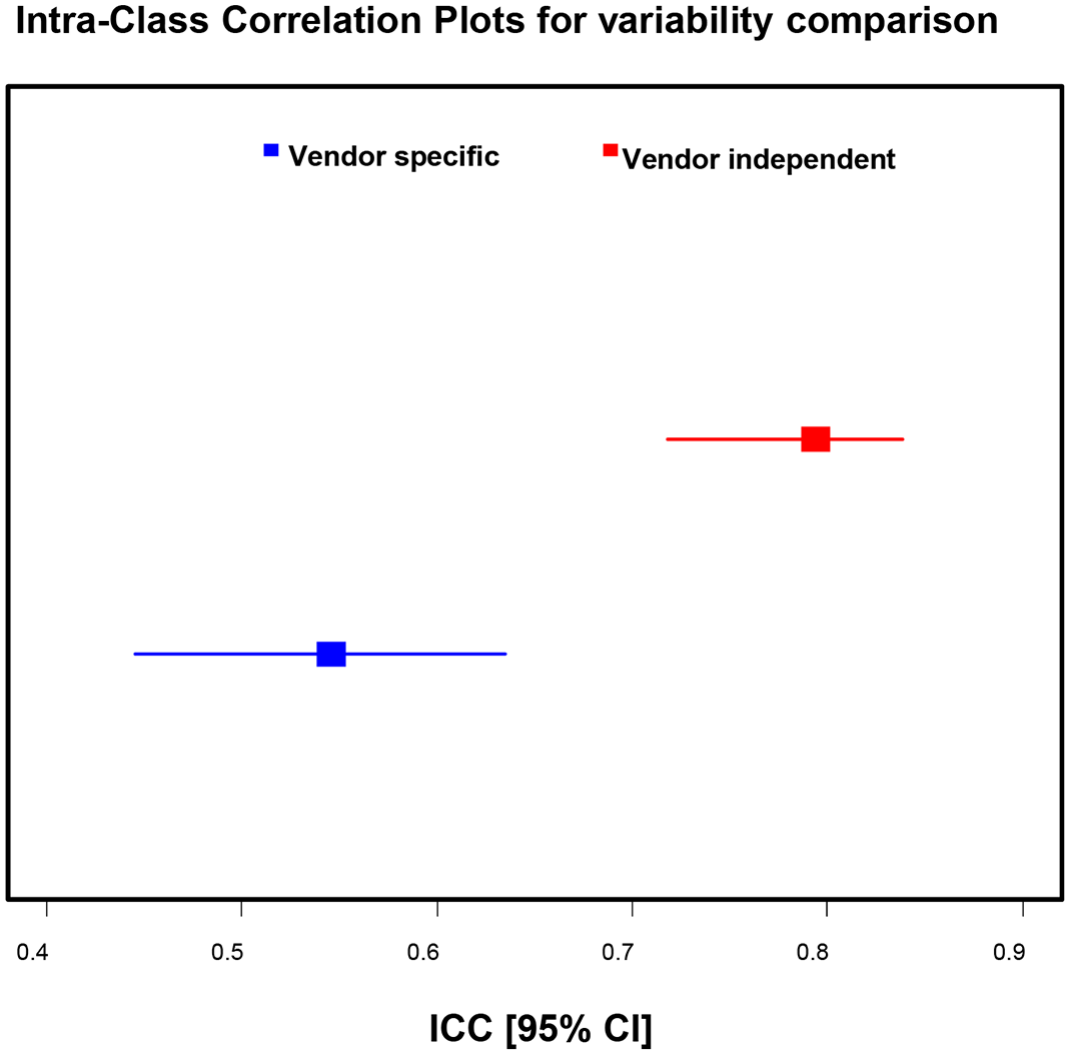
Intra-Class Correlation plots to assess the variability of strain measurements derived from vendor specific and vendor independent analyses. The plots show no overlapping between the 95% CI for ICC between the two approaches: vendor specific (blue) vs. vendor independent (red).

Inter-observer variability for the vendor-specific software was relatively good with ICC values ranging from 0.83 to 0.96 (CS) and 0.90 to 0.98 (LS) while the intra-observer reproducibility was found to range from 0.97 to 0.99 (CS) and 0.96–0.99 (LS). For the vendor-independent program, inter-observer variability ranged from ICC = 0.93 to 0.99 (CS) and 0.98 to 0.99 (LS) while intra-observer reproducibility ranged from 0.98 to 0.99 (CS) and 0.99 (LS) (Fig 6).

## Discussion

A fundamental obstacle to inter-institutional, and in some cases intra-institutional, collaboration on echocardiography-based studies is the lack of reproducibility between vendors [28, 29]. Badano *et al* concluded that longitudinal 3D-echocardiography studies must be conducted using the same imaging platform to assure accuracy [29]. Previous inter-vendor reproducibility studies have shown a statistically significant difference in 3DE-derived CS and LS values between ultrasound systems [28, 29, 35]. These findings are in agreement with the results of this study, which verify that CS and LS values determined by vendor-dependent software exhibit low levels of agreement. Recognizing the critical need for standardization in strain imaging, the European Association of Cardiovascular Imaging, EACVI) and the American Society of Echocardiography (ASE) has also published a technical document intended to create a common standard [28]. The observed lack of reliability between vendors may result from differences in tracking algorithms, interpolation techniques and strain calculations [29, 36].

The ability to convert full-volume acquisitions from multiple ultrasound systems to digital imaging and communications in medicine (DICOM) data allows the use of a single vendor-independent analysis utility [33]. In this study, the use of vendor-independent software to determine CS and LS values was shown to reduce the variability between ultrasound systems such that there was no significant difference. The ability of a vendor-independent analysis package to reduce inter-vendor variability has been shown in previous studies [28, 37].

### Clinical Significance

The use of this vendor-independent quantification technology in a clinical setting has a wide variety of beneficial applications. Previous studies have indicated that the same ultrasound system and vendor-dependent analysis package must be used for a longitudinal study to assure accuracy [30, 36, 37]. However, the results of this study suggest that the use of a vendor-independent software package obviates the variability in both CS and LS values between ultrasound systems. This technology has the potential to reduce health care costs by precluding the reacquisition of images previously captured by a different ultrasound system than the one currently in use at a particular facility. Additionally, the quality of patient care may be improved by the ability to compare previous 3DE studies to present values and subsequently determine the progression of cardiac function.

This technology could also promote an increase in inter-institutional collaboration resulting from the ability to analyze data acquired on multiple ultrasound systems. This would allow studies to be completed in shorter periods of time with greater subject diversity. Due to the increased potential patient population associated with collaborative studies, uncommon or rare disorders could be much more comprehensively studied. Additionally, the application of this vendor-independent analysis package may allow unprecedented longitudinal study of disease progression.

### Study Limitations

Temporal resolution was optimized, but not explicitly examined as a source of variability in this study. Previous studies have determined that temporal resolution values consistent with those used in this study are not a source of variability [30, 38].

The nature of this *in vitro* model prevents examination of the effects of image quality as all acquisitions occurred under optimal conditions. The proposed model of the CS in this study, however, is different with respect to the in vivo situation, as it does not keep in consideration electrical conduction, which causes a delay in contraction of different sections of the heart. Additionally, this phantom study used extrinsic pump to generate pulsatile cardiac motion, which is quite different than intrinsic myocardial contraction. Based on the results of this study and the known limitations of strain quantification reproducibility, especially associated with CS, it is important to continue this research using *in vivo* acquisitions.

This study utilized regional strain values at the mid-anterior segment due to its presence in the near field to optimize resolution. These values were used as surrogates for global strain; however, they may not accurately represent global strain values due to position relative to the transducer. This limitation in study design was necessary due to the practical constraints of using sonomicrometry crystals to determine stain in a passively driven cardiac model. Variability, therefore, could have been reduced by optimized image quality allowed in the phantom setting and/or amplified by regional evaluation.

## Conclusions

Circumferential and longitudinal regional strain values differ when quantified by vendor specific analysis packages; however, this variability is mitigated by use of a vendor independent quantification method. The results of this study suggest the plausibility of inter-institutional collaborative studies, regardless of different ultrasound systems used in acquisition.

## References

[1] Tee M, Noble JA, Bluemke DA. Imaging techniques for cardiac strain and deformation: Comparison of echocardiography, cardiac magnetic resonance and cardiac computed tomography. Expert Rev Cardiovasc Ther 2013; 11(2):221–231. 10.1586/erc.12.182 23405842

[2] Parra DA, Vera K. New imaging modalities to assess cardiac function: Not just pretty pictures. Curr Opin Pediatr 2012; 24(5):557–564. 10.1097/MOP.0b013e328357bae3 22935758

[3] Gao H, Allan A, McComb C, Luo X, Berry C. Left ventricular strain and its pattern estimated from cine CMR and validation with DENSE. Phys Med Biol 2014; 59(13):3637–3656. 10.1088/0031-9155/59/13/3637 24922458

[4] Soleimanifard S, Abd-Elmoniem KZ, Sasano T, Agarwal HK, Abraham MR, Abraham TP, et al Three-dimensional regional strain analysis in porcine myocardial infarction: A 3T magnetic resonance tagging study. J Cardiovasc Magn Reson 2012; 14:85 ; PMCID: PMC3534020 10.1186/1532-429X-14-85 23237210 PMC3534020

[5] Song CF, Zhou Q, Guo RQ. Alteration in the global and regional myocardial strain patterns in patients with inferior ST-elevation myocardial infarction prior to and after percutaneous coronary intervention. Kaohsiung J Med Sci 2014; 30(1):29–34. 10.1016/j.kjms.2013.04.005 24388056 PMC11916695

[6] Bhan A, Sirker A, Zhang J, Protti A, Catibog N, Driver W, et al High-frequency speckle tracking echocardiography in the assessment of left ventricular function and remodeling after murine myocardial infarction. Am J Physiol Heart Circ Physiol 2014; 306(9):H1371–H1383. ; PMCID: PMC4010665 10.1152/ajpheart.00553.2013 24531814 PMC4010665

[7] Bilchick KC, Kuruvilla S, Hamirani YS, Ramachandran R, Clarke SA, Parker KM, et al Impact of mechanical activation, scar, and electrical timing on cardiac resynchronization therapy response and clinical outcomes. J Am Coll Cardiol 2014; 63(16):1657–1666. ; PMCID: PMC4427624 10.1016/j.jacc.2014.02.533 24583155 PMC4427624

[8] Taylor RJ, Umar F, Moody WE, Meyyappan C, Stegemann B, Townend JN, et al Feature-tracking cardiovascular magnetic resonance as a novel technique for the assessment of mechanical dyssynchrony. Int J Cardiol 2014; 175(1):120–125. 10.1016/j.ijcard.2014.04.268 24852836

[9] Kapetanakis S, Bhan A, Murgatroyd F, Kearney MT, Gall N, Zhang Q, Yu CM, Monaghan MJ. Real-time 3D echo in patient selection for cardiac resynchronization therapy. JACC: Cardiovasc Imaging 2011; 4(1):16–26. 21232699 10.1016/j.jcmg.2010.09.021

[10] Li CH, Carreras F, Leta R, Carballeira L, Pujadas S, Pons-Llado G. Mechanical left ventricular dyssynchrony detection by endocardium displacement analysis with 3D speckle tracking technology. Int J Cardiovasc Imaging 2010; 26(8):867–870. 10.1007/s10554-010-9644-x 20711677

[11] Purevjav E, Varela J, Morgado M, Kearney DL, Li H, Taylor MD, et al Nebulette mutations are associated with dilated cardiomyopathy and endocardial fibroelastosis. J Am Coll Cardiol 2010; 56(18):1493–1502. ; PMCID: PMC2957670 10.1016/j.jacc.2010.05.045 20951326 PMC2957670

[12] Nishii T, Kono AK, Shigeru M, Takamine S, Fujiwara S, Kyotani K, et al Cardiovascular magnetic resonance T2 mapping can detect myocardial edema in idiopathic dilated cardiomyopathy. Int J Cardiovasc Imaging 2014; 30(Suppl 1):65–72. 10.1007/s10554-014-0414-z 24715436

[13] Everitt MD, Sleeper LA, Lu M, Canter CE, Pahl E, Wilkinson JD, et al; Pediatric Cardiomyopathy Registry Investigators. Recovery of echocardiographic function in children with idiopathic dilated cardiomyopathy: Results from the Pediatric Cardiomyopathy Registry. J Am Coll Cardiol 2014; 63(14):1405–1413. PMCID: PMC4030008 24561146 10.1016/j.jacc.2013.11.059 PMC4030008

[14] Ismail TF, Jabbour A, Gulati A, Mallorie A, Raza S, Cowling TE, et al Role of late gadolinium enhancement cardiovascular magnetic resonance in the risk stratification of hypertrophic cardiomyopathy. Heart 2014; 100(23)1851–1858. 10.1136/heartjnl-2013-305471 24966307

[15] Urbano-Moral JA, Rowin EJ, Maron MS, Crean A, Pandian NG. Investigation of global and regional myocardial mechanics with 3-dimensional speckle tracking echocardiography and relations to hypertrophy and fibrosis in hypertrophic cardiomyopathy. Circ Cardiovasc Imaging 2014; (1)7:11–19. 10.1161/CIRCIMAGING.113.000842 24275954

[16] Fox PR, Basso C, Thiene G, Maron BJ. Spontaneously occurring restrictive nonhypertrophied cardiomyopathy in domestic cats: A new animal model of human disease. Cardiovasc Pathol 2014; 23(1):28–34. 10.1016/j.carpath.2013.08.001 24035181

[17] Cheng H, Zhao S, Jiang S, Lu M, Yan C, Ling J, et al The relative atrial volume ratio and late gadolinium enhancement provide additive information to differentiate constrictive pericarditis from restrictive cardiomyopathy. J Cardiovasc Magn Reson 2011; 13:15 ; PMCID: PMC3058035 10.1186/1532-429X-13-15 21349202 PMC3058035

[18] Sasaki N, Garcia M, Lytrivi I, Ko H, Nielsen J, Parness I, et al Utility of Doppler tissue imaging-derived indices in identifying subclinical systolic ventricular dysfunction in children with restrictive cardiomyopathy. Pediatr Cardiol 2011; 32(5):646–651. 10.1007/s00246-011-9948-1 21442400

[19] Lee SC, Lee J, Jin DK, Kim JS, Jeon ES, Kwun YH, et al Improvement of cardiac function by short-term enzyme replacement therapy in a murine model of cardiomyopathy associated with Hunter syndrome evaluated by serial echocardiography with speckle tracking 2-D strain analysis. Mol Genet Metab 2014; 112(3):218–223. 10.1016/j.ymgme.2014.04.005 24836711

[20] Liu L, Tuo S, Zhang J, Zuo L, Liu F, Hao L, et al Reduction of left ventricular longitudinal global and segmental systolic functions in patients with hypertrophic cardiomyopathy: Study of two-dimensional tissue motion annular displacement. Exp Ther Med 2014; 7(6):1457–1464. ; PMCID: PMC4043569 24926326 10.3892/etm.2014.1617 PMC4043569

[21] Biswas M, Sudhakar S, Nanda NC, Buckberg G, Pradhan M, Roomi AU, et al Two- and three-dimensional speckle tracking echocardiography: Clinical applications and future directions. Echocardiography 2013; 30(1):88–105. 10.1111/echo.12079 23297852

[22] Wu VC, Takeuchi M, Otani K, Haruki N, Yoshitani H, Tamura M, et al Effect of through-plane and twisting motion on left ventricular strain calculation: Direct comparison between two-dimensional and three-dimensional speckle-tracking echocardiography. J Am Soc Echocardiogr 2013; 26(11):1274–1281. 10.1016/j.echo.2013.07.006 23953702

[23] Negishi K, Negishi T, Agler DA, Plana JC, Marwick TH. Role of temporal resolution in selection of the appropriate strain technique for evaluation of subclinical myocardial dysfunction. Echocardiography 2012; 29(3):334–339. 10.1111/j.1540-8175.2011.01586.x 22150476

[24] Takeuchi M, Otani S, Weinert L, Spencer KT, Lang RM. Comparison of contrast-enhanced real-time live 3-dimensional dobutamine stress echocardiography with contrast 2-dimensional echocardiography for detecting stress-induced wall-motion abnormalities. J Am Soc Echocardiogr 2006; 19(3):294–299. 16500492 10.1016/j.echo.2005.10.008

[25] Maffessanti F, Nesser HJ, Weinert L, Steringer-Mascherbauer R, Niel J, Gorissen W, et al Quantitative evaluation of regional left ventricular function using three-dimensional speckle tracking echocardiography in patients with and without heart disease. Am J Cardiol 2009; 104(12):1755–1762. 10.1016/j.amjcard.2009.07.060 19962489

[26] Streiff C, Zhu M, Panosian J, Sahn DJ, Ashraf M. Comprehensive evaluation of cardiac function and detection of myocardial infarction based on a semi-automated analysis using full-volume real time three-dimensional echocardiography. Echocardiography 2014; 32(2):332–338. 10.1111/echo.12643 24930502

[27] Luis SA, Yamada A, Khandheria BK, Speranza V, Benjamin A, Ischenko M, et al Use of three-dimensional speckle-tracking echocardiography for quantitative assessment of global left ventricular function: A comparative study to three-dimensional echocardiography. J Am Soc Echocardiogr 2014; 27(3):285–91. 10.1016/j.echo.2013.11.002 24325960

[28] Voigt JU, Pedrizzetti G, Lysyansky P, Marwick TH, Houle H, Baumann R, et al Definitions for a common standard for 2D speckle tracking echocardiography: Consensus document of the EACVI/ASE/Industry Task Force to standardize deformation imaging. Eur Heart J Cardiovasc Imag 2015; 16(1):1–11. . 25525063 10.1093/ehjci/jeu184

[29] Gayat E, Ahmad H, Weinert L, Lang RM, Mor-Avi V. Reproducibility and inter-vendor variability of left ventricular deformation measurements by three-dimensional speckle-tracking echocardiography. J Am Soc Echocardiogr 2011; 24(8):878–885. 10.1016/j.echo.2011.04.016 21645991

[30] Badano LP, Cucchini U, Muraru D, Al Nono O, Sarais C, Iliceto S. Use of three-dimensional speckle tracking to assess left ventricular myocardial mechanics: Inter-vendor consistency and reproducibility of strain measurements. Eur Heart J Cardiovasc Imaging 2013; 14(3):285–293. 10.1093/ehjci/jes184 22968525

[31] Yodwut C, Weinert L, Klas B, Lang RM, Mor-Avi V. Effects of frame rate on three-dimensional speckle-tracking-based measurements of myocardial deformation. J Am Soc Echocardiogr 2012; 25(9):978–985. 10.1016/j.echo.2012.06.001 22766029

[32] Yodwut C, Lang RM, Weinert L, Ahmad H, Mor-Avi V. Three-dimensional echocardiographic quantitative evaluation of left ventricular diastolic function using analysis of chamber volume and myocardial deformation. Int J Cardiovasc Imaging 2013; 29(2):285–293. 10.1007/s10554-012-0087-4 22752362

[33] Kaku K, Takeuchi M, Tsang W, Takigiku K, Yasukochi S, Patel AR, Mor-Avi V, Lang RM, Otsuji Y. Age-related normal range of left ventricular strain and torsion using three-dimensional speckle-tracking echocardiography. J Am Soc Echocardiogr 2014; 27(1):55–64. 10.1016/j.echo.2013.10.002 24238753

[34] Koopman LP, Slorach C, Manlhiot C, McCrindle BW, Jaeggi ET, Mertens L, et al Assessment of myocardial deformation in children using Digital Imaging and Communications in Medicine (DICOM) data and vendor independent speckle tracking software. J Am Soc Echocardiogr 2011; 24(1):37–44. 10.1016/j.echo.2010.09.018 21095099

[35] Risum N, Ali S, Olsen NT, Jons C, Khouri MG, Lauridsen TK, et al Variability of global left ventricular deformation analysis using vendor dependent and independent two-dimensional speckle-tracking software in adults. J Am Soc Echocardiogr 2012; 25(11):1195–1203. 10.1016/j.echo.2012.08.007 22981228

[36] Yuda S, Sato Y, Abe K, Kawamukai M, Kouzu H, Muranaka A, et al Inter-vendor variability of left ventricular volumes and strains determined by three-dimensional speckle tracking echocardiography. Echocardiography 2014; 31(5):597–604. 10.1111/echo.12432 25070187

[37] Marwick TH. Consistency of myocardial deformation imaging between vendors. Eur J Echocardiogr 2010; 11(5):414–416. 10.1093/ejechocard/jeq006 20164088

[38] Negishi K, Lucas S, Negishi T, Hamilton J, Marwick TH. What is the primary source of discordance in strain measurement between vendors: Imaging or analysis? Ultrasound Med Biol 2013; 39(4):714–720. 10.1016/j.ultrasmedbio.2012.11.021 23414723

